# A Review of Dietary Therapy for IBD and a Vision for the Future

**DOI:** 10.3390/nu11050947

**Published:** 2019-04-26

**Authors:** Nicole Green, Talya Miller, David Suskind, Dale Lee

**Affiliations:** Department of Pediatric Gastroenterology and Hepatology, Seattle Children’s Hospital, Seattle, WA 98105, USA; nicole.green@seattlechildrens.org (N.G.); talya.miller@seattlechildrens.org (T.M.); david.suskind@seattlechildrens.org (D.S.)

**Keywords:** inflammatory bowel disease, Crohn, ulcerative colitis, diet, nutrition, exclusive enteral nutrition

## Abstract

Inflammatory bowel disease (IBD) is a chronic inflammatory condition affecting the gastrointestinal tract. The rising incidence of IBD has been associated with urbanization and shifts toward a Westernized diet. The intestinal microbiome has been a focus of disease pathogenesis and also therapeutic intervention. Dietary therapy for IBD has been well-studied with exclusive enteral nutrition, a formula-based diet with the exclusion of foods. In addition, interest in food-based exclusion diets has been increasing, with patients and families leading the charge. Challenges with dietary therapy for IBD include the lack of understanding of a detailed mechanistic pathway to explain the impact of diet on IBD pathogenesis and the difficult nature of designing and implementing dietary clinical trials. Epidemiological studies have demonstrated associations and intervention studies have demonstrated efficacy, but specific dietary targets remain as hypotheses at present. Current IBD therapy focuses on suppression of the immune system, yet the incomplete efficacy of present drugs suggests that other therapies must be developed and employed. Dietary interventions, with known ability to modulate the intestinal microbiome, are a unique opportunity to improve outcomes in IBD. Dietary intervention trials are challenging, and capturing both broad dietary patterns as well as exposure to individual food compounds is important. With increasing patient interest and preliminary research in dietary therapy indicating efficacy, it is imperative to further advance the science of utilizing diet in IBD, as well as to support patients by proactively addressing diet within their care plan.

## 1. Background

Nutritional interventions play a central role in the treatment and management of inflammatory bowel disease (IBD). While malnutrition is a common presentation in IBD, nutritional interventions have not only focused on correcting nutrient deficiencies but also on improving disease activity and symptoms. In addition to other societies, the North American Society for Pediatric Gastroenterology Hepatology and Nutrition has issued clinical guidelines emphasizing the importance of nutritional interventions in pediatric IBD. These guidelines focus on the use of exclusive enteral nutrition (EEN), a formula-based therapy, in pediatric Crohn’s disease [1,2,3]. Since the publication of these guidelines, further research has expanded the concept of food-based nutritional intervention in patients with IBD. Recent research has shown the impact of diet on all three components of the IBD paradigm: the intestinal microbiome, mucosal integrity, and the immune system. With known influence on disease pathogenesis and perpetuation, diet in IBD is developing into a powerful, state-of-the-art treatment for IBD.

The history of nutritional therapy for IBD began with initial observations in hospitalized adults with severe Crohn’s disease who improved on EEN [4,5]. Since the initial report of EEN in IBD in the late 1970s, there have been over 200 publications on EEN with multiple meta-analyses showing that the use of EEN in children with Crohn’s disease is as effective as corticosteroids at inducing remission of active inflammation [6,7]. In adults with Crohn’s disease, EEN has shown conflicting results. A 2018 Cochrane Database Systematic Review comparing EEN versus steroids showed greater efficacy of steroids [8]. It is unclear whether the perceived difference between efficacy in pediatric and adult EEN studies is related to compliance with therapy, greater duration of disease, or prior exposures to immunosuppressive drugs. Two studies in adults with newly diagnosed Crohn’s disease showed similar efficacy with EEN involving a nasogastric tube option [9,10].

Immunosuppressive therapies are not effective at treating IBD for all patients and carry risk. The foods we eat have been hypothesized to play a role in pathogenesis as well as exacerbation of inflammation for many years. With basic science research showing the effect of dietary exposures on the intestinal microbiome as well as mucosal integrity, renewed interest has emerged on the ability of diet to treat the inflammatory burden characterizing patients with IBD. Despite the complexities and difficulty in doing clinical nutrition research, epidemiologic research has identified dietary components that are protective and risk factors for the development of IBD [11,12]. In addition, many diets have been reported to be efficacious in small case series, including the specific carbohydrate diet (SCD), IBD anti-inflammatory diet (IBD-AID), Crohn’s disease exclusion diet (CDED), and semi-vegetarian diet [13]. The SCD developed by Dr. Sydney Haas, a pediatrician, in the 1930s to treat patients with celiac disease has been one of the better studies of exclusion diets used in IBD [14]. It was popularized in the late 20th century by Elaine Gottschall, whose daughter’s ulcerative colitis was successfully treated with the SCD by Dr. Haas [15]. The SCD and other exclusion diets have risen in popularity among patients, and research efforts are underway attempting to keep pace with patient demand.

## 2. IBD Pathogenesis

The pathogenesis of IBD, while not completely elucidated, is thought to be multifactorial with interplay between genetics, the environment, and the immune system. The major environmental factors associated with IBD include shifts in diet, antibiotics, other medications, air pollution, infections, major life stressors, and smoking [12,16]. Diet is an environmental exposure that has changed over time, is modifiable, and has increasingly become a component of the treatment paradigm for IBD. A look at the epidemiologic patterns of IBD, with highest prevalence in Northern Europe and North America and rapidly increasing incidence in more-industrialized countries, has led to the theory that a Westernized lifestyle, and in particular diet, is linked to the rising incidence of IBD [17].

Understanding the role of dietary therapy in IBD requires an appreciation of the intestinal microbiome and intestinal mucosal integrity or barrier function. The intestinal microbiota consists of thousands of bacterial species with a gene pool 150 times larger than the human genome, with tremendous functional diversity [18]. The intestinal mucosal barrier consists of epithelial cells connected by tight junctions, antimicrobial factors, and overlying inner and outer mucus layers that limit the interaction between the microbiome and immune system [19]. In IBD, the homeostasis between the microbiome, intestinal barrier, and immune system is disrupted resulting in dysbiosis, increased barrier permeability, and inflammation [20].

The intestinal microbiome is highly adaptable and responsive to environmental factors. From the time of birth, environmental factors such as mode of delivery (vaginal versus caesarean section) and feeding method (breast feeding versus formula feeding) influence the microbiome [21,22]. Dietary patterns have a profound effect on the gut microbiome, and Wu et al., demonstrated that a high fat and animal protein diet compared to a high carbohydrate diet was associated with significant differences in the intestinal microbiome [23]. Numerous studies have highlighted differences in the microbiome between individuals with IBD and healthy individuals, with an overall trend towards loss of diversity of the microbiota in IBD [24,25,26,27]. A decrease in certain *Clostridium* clusters is a consistent finding among studies [20,25]. These species stimulate regulator T cells, resulting in immune tolerance and reduced gastrointestinal inflammation [20,28]. Conversely, members of the family Enterobacteriaceae are consistently elevated in IBD [20]. These include the well-known intestinal pathogens *Campylobacter* spp., *Salmonella* spp., and *Escherichia coli*. Whether these patterns are a cause or consequence of intestinal inflammation remains debatable. In the healthy state, the microbiota comes into contact with epithelial cells and the immune system in a highly controlled manner [29]. In IBD, the mucosal barrier is compromised, resulting in translocation of the intestinal microbiota and resultant immune system activation and inflammatory response [30].

## 3. Scientific Basis of Dietary Therapy for IBD

Many studies have evaluated the ability of diet to modulate intestinal microbiota and influence epithelial barrier function. Low fiber diets have been linked to IBD with a posited mechanism of reduction in short-chain fatty acid production by commensal bacteria whose preferred energy source is fiber [31]. Butyrate, a short-chain fatty acid, is essential for colonic health and the major energy source for colonocytes [32]. In addition to supporting intestinal barrier function, short-chain fatty acids also promote immune tolerance by promoting development of T-regulatory cells [33,34]. Food additives are commonly consumed by patients with IBD, and specific dietary emulsifiers (carboxymethylcellulose and polysorbate 80) have been shown to induce low grade inflammation and metabolic syndrome in wild type mice and promote colitis in genetically predisposed IL-10 knockout mice [35,36]. The emulsifiers perturbed the host microbiota, resulting in increased inflammatory potential with a rise in the number of mucolytic bacteria, and erosion of the protective mucous layer. At present, efforts are underway to better evaluate the frequency of exposure to food additives and the effect of these compounds on the intestinal system.

A summary of clinical trials and data reporting on the outcomes of dietary therapies in IBD are well described in greater detail elsewhere [3,37,38,39]. Importantly, many of these trials are smaller in size, deemed to produce a lower grade of evidence, and limited by the lack of long-term outcome data. In a recently published Cochrane review, Limketkai et al. analyzed 18 randomized controlled trials, comprising 1878 participants, published between 1965 and 2018 [37]. The intervention diets involved complete exclusion or significant limitation of one or more food groups. Diets included low refined carbohydrates; low microparticles; low calcium; low red and processed meat; low disaccharides, grain, saturated fats, red, and processed meat; symptoms-guided diets; highly-restricted organic diet; milk-free; Alberta-based anti-inflammatory diet; and Carrageenan-free diet. The different studies looked at various outcomes, including induction of remission, clinical relapse, surrogate biomarkers of inflammation, endoscopic improvement, health-related quality of life, and the need for surgery. This review concluded that no firm conclusions could be reached about the effect of these dietary interventions on IBD.

Though these included studies may be superior in that they are randomized controlled studies, additional studies on EEN and broader restrictions diets have shown clearer impact on IBD [40,41,42]. A key distinction from the randomized trials is the more profound nature of dietary intervention with EEN and many restrictions diets. Though not all controlled studies, the findings in these numerous, heterogeneous studies suggest the impact of dietary interventions on inflammation and clinical outcomes. Together with strong patient desire for dietary guidance and the opportunity for greater efficacy with conventional immunosuppression, larger more definitive studies are needed.

The most rigorously studied dietary interventions in IBD is exclusive enteral nutrition (EEN), a formula-based therapy for Crohn’s disease. Numerous studies in children and adolescents have demonstrated the ability of EEN to induce remission of active Crohn’s disease in 80–85% of patients [43,44]. EEN is equivalent to corticosteroid therapy in inducing clinical remission and superior at achieving endoscopic mucosal healing [43,45]. EEN is a first-line therapy for pediatric Crohn’s disease in many parts of the world, and the treatment protocol typically involves administration of formula to provide 100% of caloric needs and exclusion of food for 6–8 weeks [1].

The exact mechanism by which EEN exerts its effect is unknown. Hypothesized mechanisms (Table 1) include limiting antigen exposure, antigenic monotony, enhancing nutritional status and nutrient delivery, altering the microbiome and immune response, and avoidance of deleterious compounds [3,5,20,46]. As EEN and exclusion diets are extremely different interventions, it is likely that the mechanism by which they impact disease is likewise different. Despite disease activity improvement, studies looking at fecal metagenomics in children with Crohn’s disease have found that EEN seems to decrease gut microbiota diversity and promote a more “dysbiotic” state when compared to healthy controls [47]. Microbial functional capacity also decreased with EEN as did genes encoding proteins involved in B-complex vitamin biosynthesis [47]. In studies evaluating changes to the intestinal microbiome in patients with IBD treated with conventional medical therapies, dysbiosis improved with therapy [48]. Clearly therefore, the relationship between the beneficial effects of EEN and alterations in the gut microbiota needs further characterization and may result from alterations in beneficial or harmful metabolites produced by the bacteria.

EEN established the ability of diet to drive Crohn’s disease into remission, but EEN is difficult to sustain as long-term maintenance therapy and is not effective for ulcerative colitis. Exclusion diets, though, are practical as long-term therapy and have been found to be useful in both Crohn’s disease and ulcerative colitis. One of the most studied exclusion diets is the specific carbohydrate diet (SCD). This diet removes all grains, sweeteners (except for honey), processed food, and all milk products except for hard cheeses and yogurt fermented longer than 24 h. Both clinical and laboratory improvement have been reported in pediatric and adult patients with IBD [49,50,51,52]. One study over 12 weeks in children and adolescents used capsule endoscopy and demonstrated mucosal healing [53]. Therapy with the SCD has been shown to result in significant changes in microbial composition [49]. The SCD has been the best-studied food-based diet for IBD, and one survey with 417 respondents found patients reported significant improvement in symptoms, but that only 17% of patients partnered with a healthcare professional in the implementation of the SCD [50].

The Crohn’s disease exclusion diet (CDED) is based on the hypothesis that components of the Western diet promote a pro-inflammatory microbiome and may disrupt the mucosal barrier. The diet focuses on excluding gluten, dairy, gluten-free baked goods, animal fat, emulsifiers, and all canned or processed foods. A prospective cohort of pediatric and adult participants with mild to moderate Crohn’s disease treated with partial enteral nutrition (formula providing approximately 50% of daily calories) and the CDED showed success in achieving induction of clinical remission [41]. The IBD anti-inflammatory diet (IBD-AID) is derived from the SCD. It is a whole foods-based diet that restricts the intake of complex carbohydrates such as refined sugar, gluten-based grains, and certain starches from the diet, but also incorporates ingestion of prebiotics and probiotics. The diet also incorporates phases of food textures. In a small retrospective case series of patients with IBD on the IBD-AID for at least 4 weeks, all demonstrated improvement in clinical symptoms [54].

In a study of a semi-vegetarian diet in patients with remission of Crohn’s disease induced by either medical therapy or surgery, patients maintained a greater rate of clinical remission over 2 years [55]. The CD-TREAT diet hypothesizes that a whole-food diet based on the composition of Modulen^TM^, a commonly utilized formula for EEN in Europe, can emulate the efficacy of EEN [56]. In the component of the study evaluating healthy adults, similar microbiome and metabolome changes were seen with both the CD-TREAT diet and EEN. In the part of the study evaluating five children with active Crohn’s disease, the CD-TREAT diet resulted in a significant decline in fecal calprotectin, though this surrogate marker of intestinal inflammation remained elevated at both 4 and 8 weeks.

The worsening of dysbiosis and decrease in butyrate production demonstrated with EEN therapy is seemingly counterintuitive, as is the lack of any fiber content in the formulas commonly utilized for EEN [57,58]. It may be the case that EEN acts via a unique mechanism of action to impact inflammation in IBD in comparison to the action of restrictions diets. As the gut microbiome can both drive inflammation and respond to underlying inflammation, further elucidation of the complex interaction between diet, microbiome, and host will help guide future therapy. With the paradoxical findings seen in EEN versus whole food restriction diets, changes occurring while transitioning from one diet to another may provide unique insights into mechanisms.

Epidemiologic studies have demonstrated greater risk of developing IBD with increased intake of total fat, polyunsaturated fatty acids, omega-6 fatty acids, and meat, while fruits, vegetables, and fiber intake have been shown to have protective effects [11]. Greater meat intake has been associated with both increased risk of ulcerative colitis relapse in adults and decreased rate of achieving remission of Crohn’s disease in children on partial enteral nutrition therapy [59,60]. Conversely the only existing randomized study on red meat showed no association between red meat or processed meat consumption and risk of Crohn’s disease relapse, as measured by self-reported symptoms [61]. A limitation of these studies evaluating individual dietary components is the potential for confounding factors due to numerous associations between individual foods consumed [62].

While some dietary interventions have demonstrated impacts on inflammation, other interventions, such as the low FODMAP diet, have been demonstrated to impact functional gastrointestinal symptoms in patients with IBD [63,64,65]. A variety of exclusion diets have shown efficacy at treating inflammation in small case series reports, but additional studies are needed to better substantiate these findings. While these studies suggest that specific food components may be deleterious, it may be the complex interactions of food components within the food matrix with the intestinal microbiome that trigger and perpetuate the cycle of inflammation in IBD. At present, the mechanism by which dietary interventions impact inflammation in IBD remains unknown. Studies involving the microbiome, metabolome, and proteome are beginning to shed insight, and will help guide the movement to more targeted dietary therapeutic approaches in the future. The mechanism of action for dietary therapies requires further study in both clinical and preclinical models.

## 4. Contrasting Pharmacological and Dietary Therapy

The goals of IBD therapy include resolution of symptoms, mucosal healing, improved quality of life, nutritional restitution, and minimizing adverse effects associated with therapy. For both nutritional therapy and conventional medical therapy, disease remission is often a singular focus, but the broader goals of the patient are important in the discussions about therapy. Primary outcomes in dietary and drug trials for IBD, though, should be the same, with a focus on evaluation of objective markers of inflammation, including labs, imaging, endoscopic findings, and surrogate markers of intestinal inflammation. An ongoing challenge for dietary trials is the ability to fund trials that are not sponsored by corporations with a financial incentive to drive development of a new therapeutic.

EEN in pediatric Crohn’s disease has been demonstrated to lead to high rates of remission (85%) and superior mucosal healing when compared to treatment with corticosteroids [40,43,66]. Immunosuppressive therapy for IBD with monoclonal antibody anti-TNF-alpha drugs (e.g., infliximab or adalimumab), with or without concomitant immunomodulatory drugs are widely viewed as one of the most efficacious approaches to inducing clinical remission. A randomized-controlled trial in adults with newly diagnosed Crohn’s disease demonstrated steroid-free remission at week 26 for 56.8% of patients on infliximab plus azathioprine, and 44.4% for those on infliximab alone [67]. A trial of infliximab in children demonstrated clinical remission at week 10 in 58.9% of participants, and 55.8% at week 54 [68]. In the ACT1 and ACT2 trials of infliximab in adult ulcerative colitis, clinical remission at week 8, 30, and 54 was never above 40% [69]. Similarly, a pediatric trial of infliximab in ulcerative colitis showed week 54 remission in less than 40% of study participants [70]. These data suggest that medications, though effective, do not induce remission in all patients with IBD, and room exists to further optimize therapy with newer medical therapies, and dietary interventions.

Like drugs, dietary therapy is not effective for all patients, and future personalization of dietary therapy is an opportunity. Dietary therapy can be utilized as stand-alone monotherapy, or diet can also be utilized concomitant with immunosuppressive drugs. A study in Japan evaluating the utility of partial enteral nutrition therapy (greater than 900 kcal/day from enteral nutrition with formula) with infliximab demonstrated a significant decrease in disease recurrence over the course of nearly 2 years [71]. Patients commonly employ dietary modifications to address symptoms but also attempt to control inflammation with diet [72]. Implementation of dietary changes is often guided by individual research and anecdotal experience, and the majority of patients are not guided by their medical team to involve diet as part of their IBD therapy plan [13]. At present the push to study and employ dietary measures is strongly driven by patients, while many medical practitioners remain skeptical and poorly informed about dietary interventions. As an example, despite overwhelming evidence for the use of EEN in pediatric CD, U.S. physicians still use EEN to a much lesser extent than their European and Canadian colleagues [73].

That patients and families will employ dietary therapy despite the rigor this involves suggests that tangible benefits exist. Given that therapies may be intensive for weeks to months (e.g., EEN), or require changes to dietary patterns long-term, the presence of family and community support can be key in the success of the patient’s ability to adhere to the treatment. There has been limited data published on the patient experience while on EEN; however, a study of 29 families of patients with CD who chose EEN as induction therapy showed 59% of patients would repeat EEN if needed. However, 72% of parents would be more interested in a solid food based exclusion diet option [74].

There is generally a lesser degree of concern regarding the safety of nutritional therapy when compared to immunosuppressive drug therapies (Table 2). However, potential side effects can include nutritional inadequacy, psychosocial impact, and a potential for loss to medical follow-up. Psychosocial impact in the setting of nutritional therapy was evaluated by the measure of quality of life (QOL) in children and adolescents in a trial over 8 weeks with anti-TNF therapy, partial enteral nutrition, and exclusive enteral nutrition therapy [60]. This study demonstrated that children with Crohn’s disease who achieved clinical remission on any of the three intervention arms had a significant increase in the quality of life measured by the IMPACT-III questionnaire, a validated disease-specific measure of QOL in pediatric IBD. This study evaluated sub-domains of QOL and found that body image-associated QOL was superior in the EEN group compared to the anti-TNF therapy group. Exclusion diets can be isolating and difficult to maintain, and certain patients may not be a good fit for dietary therapy. For those who demonstrate interest in dietary therapy, close monitoring, support of their family and friends, and partnership with the broader medical team (including physician, dietitian, and psychologist) helps with maintaining compliance and ensuring appropriateness of an ongoing dietary approach to IBD.

Since dietary therapies can be employed without physician prescription, there is potential for loss of medical follow-up. Without partnership with the medical team, dietary therapy may result in inadequate nutritional intake and a lack of appropriate objective evaluation of clinical, laboratory, and mucosal remission. When considering potential side effects of nutritional therapy, medical professionals must acknowledge that many patients are on dietary therapy as treatment for their IBD without formal medical guidance [50]. At a minimum, the medical team should engage patients in discussion about their dietary patterns, understand the basics of commonly utilized IBD exclusion diets, and partner with patients to closely monitor nutritional status and disease activity. Medical therapies require formal prescription from a healthcare professional and insurance provides coverage, or at least provides coverage of alternative medications. At present, dietary therapy for IBD is not broadly reimbursed by insurance. Further data on efficacy of exclusion diets is needed, but even for established therapy like EEN for Crohn’s disease, insurance companies do not provide reimbursement in the United States.

Despite the many challenges around utilizing dietary therapy for IBD, dietary interventions are eagerly sought and employed by patients. The desire to avoid or lessen the degree of immunosuppression or supplement existing medical therapies are common drivers. Given the burden associated with dietary modification when considering cost, time for food preparation, and dietary restriction, it is important to recognize that nutritional therapy may not be an appealing option for many patients. For these reasons, not all patients and families will have equitable access to or interest in nutritional therapies. No studies have yet shown the variation in average costs between the different dietary interventions.

## 5. Vision for the Future of Diet and IBD

Dietary trials present challenges, but the ability to use nutrition to treat disease is a unique opportunity. It has long been accepted that dietary exposures are associated with risk of developing IBD, but diet as therapy has not been fully integrated into the broader paradigm of IBD therapy (Table 3). Diet represents a fundamental aspect of daily living and has infinite variability; diet is a potential target for profound intervention. Dietary intervention with EEN or even a shift in fat and fiber consumption results in a tangible shift in the intestinal microbiome [23,47,75]. Efforts have been placed into the development of therapeutic approaches to selectively modulate the intestinal microbiome, including prebiotics, probiotics, antibiotics, and fecal transplantation, but persistent shaping of the microbiome requires ongoing maintenance of these therapies [76]. Dietary therapies that reshape patterns of food consumption may alter exposures to deleterious substances such as select food additives, directly influence the intestinal microbiome, and also have a direct impact on the functioning of the host immune system [3].

Many individuals institute dietary changes based on varied sources of information and also as a reaction to the onset of IBD symptoms [13,77]. Recommendations are of variable validity, and better data on the role of diet in the setting of active IBD is needed. The data on EEN therapy has led to the utilization of this as standard therapy for induction of remission in pediatric Crohn’s disease. The limitation of EEN is the difficulty in sustaining the intervention. Food-based IBD trials can involve exclusion, supplementation, or dramatic changes in dietary pattern [62]. Thus far, it appears unlikely that focused exclusion or supplementation have a role in IBD therapy, but more profound changes in dietary pattern have shown promise. Though individual dietary components may be deleterious (such as refined sugars, high fat, and specific food additives), it may be the case that the effect of these components are balanced or attenuated by other components (e.g., fiber, antioxidants, bioactive molecules). As such, we suggest that future dietary trials in IBD should consider the global dietary pattern, while also considering the role of individual dietary components. At present, published trials for exclusion diets in IBD often include a description of the general principles of the diet, but do not further characterize the variability within the proposed intervention and the impact this has on clinical outcomes. Challenges to consider in food-based dietary trials in IBD include issues with participant compliance, the measurement of compliance, and variability within a particular dietary intervention.

Though randomized controlled trials are the gold standard for the generation of evidence to drive clinical practice, innovative study design such as n-of-1 studies, and data from larger cohort studies, together with preclinical data, will also help to better substantiate the existing literature supporting dietary therapies in IBD. The paradigm of relying only on data from controlled trials is challenging in the setting of dietary interventions requiring profound changes to habitual diet, the need to maintain compliance, and the cost of implementing such trials.

As diet can be a primary or adjunctive therapy, the magnitude of effect considered as significant may be different for dietary trials as compared to drug trials. For example, if a dietary intervention results in lack of endoscopic mucosal healing but significantly decreases inflammatory burden as measured by biochemical markers, this may suggest that the intervention has a promising role as an adjunct to conventional immunosuppressive therapy. As the mechanism by which dietary therapy impacts IBD involves gut luminal exposures, the microbiome, and triggering of the mucosal immune system, diet has the potential to be utilized alongside medications to improve outcomes and durability, and minimize risks associated with immunosuppression. Drugs are not commonly brought to market with known partial efficacy, and they are not tested as frequently as adjuncts to existing therapies, but this is different for dietary therapy. Dietary trials demonstrating complete remission or even some response to therapy may have utility, as the cost and side-effect profile of dietary therapy is generally favorable for dietary therapy.

For novel pharmacologic therapeutics, often the initial identification and targeting of a specific molecular pathway guides drug development. The mechanism by which diet impacts IBD has yet to be fully elucidated [38,78]. Though the microbiome and specific deleterious substances have been identified, as of yet no definitive molecular pathway target exists for dietary therapy. At present, basic scientific research is ongoing to better elucidate mechanisms of action for both EEN and exclusion diets on treatment of active IBD, but empiric clinical trials are being pushed forward as well. Unlike drugs, with guidance, diet can be modulated within the bounds of safety, balance, and nutritionally-adequacy. Empiric dietary changes guided by experience may have value, but systematic evaluation of dietary interventions with trials is needed to produce the data to demonstrate efficacy beyond anecdote. Publication bias in favor of positive studies and poorly designed clinical studies are a limitation to the existing data for dietary therapy in IBD and need to be better addressed moving forward. A variety of exclusion diets have been proposed as therapy for IBD, and with systematic evaluation these trials will inform pre-clinical research, which in turn will help guide future trials.

Close partnership between the patient, gastroenterologist, and dietitian is necessary when utilizing dietary therapy to treat IBD. The discussion about the concept of integrating dietary therapy, goals of therapy, concerns, and a close monitoring plan should begin early. In some situations, diet will be used as monotherapy and it is of particular importance that close follow-up and monitoring of objective markers of inflammation occur, so as to prevent disease progression, select nutrient deficiencies, or global malnutrition. For physicians, the most common IBD treatment paradigms have dismissed the role of diet. A partnership with acknowledgement of the need for further clinical trial data, inherent uncertainty of efficacy for all IBD therapies, and the potential for benefit with dietary interventions will help guide progress towards better understanding of the utility of dietary therapy for individuals with IBD.

## Figures and Tables

**Table 1:** Hypothesized Mechanisms of Action for Diet Therapy in Inflammatory Bowel Disease (IBD).

Category of Mechanism	Hypothesized Mechanism	EEN	Exclusion Diets
Mechanical/physical	Liquid nutrition	x	
	Alteration of gut motility	x	x
	Gut rest	x	
Nutritional status	Improved nutrient delivery	x	
	Improved caloric delivery	x	
Epithelial barrier	Decreased permeability	x	x
	Restitution of intestinal barrier	x	x
	Improved delivery of fiber (SCFA * production)		x
Immune system	Limited antigen exposure	x	
	Antigenic monotony	x	
	Direct anti-inflammatory effect	x	x
	Alteration in bile acids	x	x
Microbiome	Shift of gut microbiome	x	x
	Stabilize gut microbiome	x	x
Specific avoidances	Avoidance of food additives		x
	Avoidance of deleterious food substances	x	x
Anti-inflammatory effect	Provide beneficial substance	x	x
	Increased antioxidant consumption		x

* SCFA: short-chain fatty acid.

**Table 2:** Comparison of Dietary Therapy and Drug Therapy.

	Dietary Therapy	Drug Therapy
Driven by patient/family interest	++	
Ability to personalize	++	+
Directly impacts quality of life *	++	
Importance of family support	++	+
Challenges with compliance	++	+
Challenges with study design	++	
Risk of nutrient deficiency	++	
Works in combination with other therapies	++	++
Physician-driven		++
Targets known pathway		++
Greater risk of infection		++
Greater risk of cancers		++
Insurance coverage		++
Partnership with large industry to study **		++

* Independent of disease activity. ** Formula companies have supported the design and implementation of some studies on EEN, but the majority of dietary trials do not have corporate backing.

**Table 3:** Dietary Therapy for IBD: Now and the Future.

**Current Accepted Place of Dietary Therapy**
EEN in pediatric Crohn’s disease Dietary therapies for symptom control Nutritional therapy to support malnutrition
**Major Areas Requiring Research**
Impact of dietary therapies with/without concomitant immunosuppression Impact of dietary therapies long-term Mechanism of action for dietary therapies Exclusion diets—dietary components of importance Cost-benefit analysis of dietary interventions Targeted dietary approaches to selectively modulate microbiome
